# Bidirectional Regulation of GABA_A_ Reversal Potential in the Adult Brain: Physiological and Pathological Implications

**DOI:** 10.3390/life14010143

**Published:** 2024-01-19

**Authors:** Haram R. Kim, Marco Martina

**Affiliations:** 1 Department of Neuroscience, Feinberg School of Medicine, Northwestern University, 300 E. Superior, Chicago, IL 60611, USA; haram.kim@northwestern.edu; 2 Department of Psychiatry, Feinberg School of Medicine, Northwestern University, 300 E. Superior, Chicago, IL 60611, USA

**Keywords:** transporter, disease, brain oscillations, excitation, inhibition

## Abstract

In physiological conditions, the intracellular chloride concentration is much lower than the extracellular. As GABA_A_ channels are permeable to anions, the reversal potential of GABA_A_ is very close to that of Cl^−^, which is the most abundant free anion in the intra- and extracellular spaces. Intracellular chloride is regulated by the activity ratio of NKCC1 and KCC2, two chloride-cation cotransporters that import and export Cl^−^, respectively. Due to the closeness between GABA_A_ reversal potential and the value of the resting membrane potential in most neurons, small changes in intracellular chloride have a major functional impact, which makes GABA_A_ a uniquely flexible signaling system. In most neurons of the adult brain, the GABA_A_ reversal potential is slightly more negative than the resting membrane potential, which makes GABA_A_ hyperpolarizing. Alterations in GABA_A_ reversal potential are a common feature in numerous conditions as they are the consequence of an imbalance in the NKCC1-KCC2 activity ratio. In most conditions (including Alzheimer’s disease, schizophrenia, and Down’s syndrome), GABA_A_ becomes depolarizing, which causes network desynchronization and behavioral impairment. In other conditions (neonatal inflammation and neuropathic pain), however, GABA_A_ reversal potential becomes hypernegative, which affects behavior through a potent circuit deactivation.

## 1. Introduction

The balance between excitation and inhibition (E/I balance) is critical for maintaining optimal function of the brain [1,2,3,4]. At the cellular level, a range of inhibitory mechanisms are designed to precisely control the excitation so that the neurons convey accurate information through the network [5,6,7,8]. In the brain, GABA (γ-aminobutyric acid) is the primary inhibitory neurotransmitter for both long-range and local inhibitory transmission. E/I imbalance can be caused by alterations of excitation, inhibition, or both. Altered E/I balance has been suggested as a causal factor for many symptoms associated with neuropsychiatric diseases, including fragile X syndrome [9], Alzheimer’s disease [10], and schizophrenia [11,12], and in most cases, it is attributable to altered GABAergic signaling [13,14].

In the brain, GABA exercises its effects by acting on two major types of receptors, the ionotropic (GABA_A_R) receptor and the metabotropic (GABA_B_R) receptor. In contrast to the GABA_B_ receptors, which set the basal tone of inhibition through indirectly opening potassium channels via G-protein activation, GABA_A_ receptors immediately alter the ion permeability of the membrane. When the GABA_A_R is activated, monovalent anions (mostly chloride but also nitrate and bicarbonate ions [15]) pass through the transmembrane channel structure of receptors. This movement of anions generates the postsynaptic current (PSC). The PSC amplitude depends on the number and availability of receptors and the intracellular concentration of anions, as well as the amount of GABA released from presynaptic terminals. Although multiple mechanisms regulate signaling through GABA_A_ receptors, including the modulation of the subunit composition of GABA_A_ receptors and the alteration in GABA metabolism, an increasing amount of work suggests that in most pathological conditions, the main alterations in GABA_A_ function are caused by changes in the relative activity of NKCC1 and KCC2, two chloride/potassium cotransporters that import and export chloride, respectively, and, thus, set the reversal potential of the current.

Over the past decades, it has become clear that despite its primary role as an inhibitory signal, the GABA_A_ current can also be excitatory. Apart from the well-known role of depolarizing GABA_A_ in developmental stages, which has been extensively investigated, numerous pathological states are associated with the reappearance of a depolarizing effect of GABA in the adult brain. Additionally, we recently showed that alterations leading to hypernegative GABA_A_ reversal potential also have an important functional impact on the adult cortex [16]. Here, we review the bidirectional regulation of GABA_A_ reversal potential in pathological conditions of the adult brain and discuss how the altered GABA_A_ function may impact network function.

## 2. GABA_A_ Reversal Potential

### 2.1. GABA_A_ Reversal Potential and Intracellular Chloride

In physiological conditions, the concentration of intracellular chloride ([Cl^−^]_i_ = 5~30 mM) is considerably lower compared to extracellular chloride ([Cl^−^]_o_ = ~140 mM) [17]. As GABA_A_ channels are permeable to anions, the reversal potential of GABA_A_ is very close to that of Cl^−^, which is the most abundant free anion in physiological solutions. A few studies have measured [Cl^−^]_i_ directly in physiological conditions using the visualization of chloride ions [18,19], but their reliability in assessing absolute chloride concentrations remains questionable. The most reliable method to estimate [Cl^−^]_i_ remains the measurement of GABA_A_ current reversal potential. The reversal potential of GABA_A_ can be obtained by measuring the GABA_A_ currents at different membrane voltages. Using the perforated patch clamp recording technique, which preserves the native concentration of anions, [Cl^−^]_i_ is calculated using the Nernst equation from the measured reversal potential.

At reversal potential, the net Cl^−^ flux across the membrane is zero, and the effect of GABA_A_ activation is entirely due to shunting inhibition (the conductance increase that diminishes cellular excitability due to Ohm’s law). In most neurons of the adult brain, the GABA_A_ reversal potential is slightly more negative than the resting membrane potential, which makes GABA_A_ hyperpolarizing. It is important to stress that because, in most cases, the GABA_A_ reversal potential is close to the resting potential, even small changes in GABA_A_ reversal can shift the effect of GABA_A_ activation from hyperpolarizing to depolarizing.

Thus, small increases in [Cl^−^]_i_ can cause the GABA_A_ current reversal potential to become depolarized relative to the resting potential. However, depolarizing reversal potential is not synonymous with an excitatory effect. It is only when this depolarizing effect exceeds the inhibitory shunting effect that GABA_A_ becomes excitatory. Due to the vicinity, in most neurons, between the value of GABA_A_ reversal potential and resting membrane potential, the opposite case is also true, and decreased [Cl^−^]_i_ boosts the influx of negatively charged ions and, therefore, strengthens the inhibitory effect of the current.

Interestingly, the internal chloride concentration in neurons is differentially regulated across developmental stages and cell types, even in the normal brain. This sets the GABA_A_ current clearly apart from the currents mediated by ionotropic glutamate receptors (AMPAR and NMDAR), which have reversal potentials (~0 mV) that are much less sensitive to changes in sodium (Na^+^) or potassium (K^+^) concentration and never change from excitatory to inhibitory. This “flexibility” of the GABA_A_ current, which is regulated by the level of [Cl^−^]_I_, sets GABA_A_ apart from other signaling systems.

### 2.2. Chloride/Potassium Cotransporters

Neuronal [Cl^−^]_i_ is primarily set by the activity of NKCC1 and KCC2, two chloride/potassium cotransporters that act in opposite directions. While NKCC1 increases [Cl^−^]_i_, KCC2 promotes Cl^−^ extrusion [20]. The actions of these two transporters are differently balanced in different locations and conditions and depend on transporter density and distribution as well as modulation (such as phosphorylation).

NKCC1 is widely expressed in cells within and outside the brain. NKCC1 transcripts and proteins are also present in non-neuronal cells like oligodendrocytes, microglia, and astrocytes [21]. KCC2 expression, on the contrary, is neuron-selective across numerous CNS structures, including the spinal cord, retina, neocortex, and most subcortical structures [22,23,24].

The structures of both transporters were well described [25,26,27,28,29]. NKCC1 cotransports Na^+^ and K^+^ inwardly together with two Cl^−^, thus increasing [Cl^−^]_i_. In contrast, KCC2 exports K^+^ and Cl^−^ and thus decreases [Cl^−^]_i_. As in mature neurons of the adult brain the activity of KCC2 exceeds that of NKCC1, [Cl^−^]_i_ is maintained at low levels, which results in the GABA_A_ current being inhibitory and hyperpolarizing.

Increased NKCC1 activity, on the other hand, results in a depolarizing shift in the GABA_A_ current reversal potential by elevating [Cl^−^]_i_. In this case, the reversal potential of the GABA_A_ current becomes depolarized compared with the resting membrane potential (see Section 2.1). In contrast, increased KCC2 activity lowers [Cl^−^]_i_ and hyperpolarizes GABA_A_ reversal potential to values considerably lower than the resting membrane potential, producing a stronger inhibitory effect.

## 3. Regulation of GABA_A_ Reversal Potential in Physiological Conditions

Depolarizing GABA_A_ has been discovered and intensively studied in immature neurons for over 30 years, and it is evolutionally conserved across vertebrate species and in *Caenorhabditis elegans* [30]. Ben-Ari (2002) suggested that there are several potential biological advantages to having GABA mediate excitation in immature neurons because, compared with the excitatory glutamatergic current, the depolarizing GABA current could be less excitotoxic for postsynaptic neurons and more energetically efficient as it does not require pumping ions against large transmembrane gradients. Strong evidence also supports the idea that the reversal potential of the GABA_A_ current is heavily regulated, even in a normally functioning adult brain, which suggests the idea that the flexibility of the GABA_A_ current effect is exploited as a general mechanism to that allows precise regulation of synaptic transmission. As there are excellent published reviews on the GABA_A_ shift in developmental stages [17,31,32], in this section, we will only briefly summarize it and focus on the GABA_A_ reversal potential regulation in the adult brain.

### 3.1. Developmental Regulation

The presence of depolarizing GABA_A_ current is the norm in the brain during early development when GABA_A_ is the main fast excitatory neurotransmitter and key to establishing normal circuitry [33,34]. Neonatal neurons express a substantial amount of NKCC1, while KCC2 expression is very low. At this stage, GABA_A_ is depolarizing, as [Cl^−^]_i_ is 20–30 mM, which implies a GABA_A_ reversal potential of −40 to −50 mV [17], while the resting potential is similar to that of mature neurons (~−70 mV) [35]. This depolarizing GABA_A_ has a critical role in synaptic formation and in the establishment of the neuronal network. GABA_A_ currents become inhibitory around postnatal Day 12 in rodents [36] and around 2 years of age in humans, largely due to increased KCC2 expression leading to lower intracellular Cl^−^ [37,38,39,40].

### 3.2. Cell Type and Spatial Heterogeneity of Chloride Transporters

In the adult brain, NKCC1 is strongly expressed in all types of non-neuronal cells—oligodendrocytes, microglia, and astrocytes [21,41,42]. It is suggested that its splice variants (NKCC1a/1b) are involved distinctively in the localization of NKCC1. NKCC1a is predominant in glial cells, and NKCC1b is found in neurons [21].

The presence of NKCC1 in glial cells increases the complexity of GABA_A_ signaling. Astrocytes affect the activity of transporters by changing the extracellular potassium and chloride concentration around the neurons [43,44]. Additionally, astrocytes directly contribute to synaptic transmission by releasing glutamate and other modulators in an activity-regulated calcium-dependent or -independent fashion [45,46,47]. Beyond the effect on membrane potential, astrocytic NKCC1 has been shown to regulate mitochondrial calcium levels in astrocytes [48]. So, NKCC1 regulation in non-neuronal cells has a major impact on brain function. A discussion of these effects is beyond the scope of this paper; interested readers can find detailed reviews on this topic [49,50].

Even when restricting the analysis to neuronal expression, changes in NKCC1/KCC2 activity are typically confined to select brain areas. For example, in a pharmacological model of cognitive impairment associated with schizophrenia, NKCC1 is increased in the ventral but not in the dorsal subregion of the medial prefrontal cortex (mPFC) [51], and in a rodent model of neuropathic pain, KCC2 expression is increased in the mPFC but not in the neighboring anterior cingulate cortex [16]. NKCC1 and KCC2 can also be differentially regulated in different layers within the same brain areas, as shown in mouse granule cells and outer and middle molecular layers following entorhinal denervation [52].

Interestingly, in the adult mouse hippocampus, the reversal potential of the GABA_A_ current in parvalbumin-positive interneurons is sensitive to KCC2 and NKCC1 blockades in a similar way to in pyramidal neurons, yet it is ~7 mV more depolarized than in pyramidal neurons [53], suggesting the differential expression of transporters between these cell types. Together with the observation that non-neuronal cells abundantly express NKCC1, these data point to the necessity of single-cell resolution studies when evaluating NKCC1/KCC2 and show that using tissue-level approaches, such as Western blot analysis of homogenized brain tissue, may easily miss cell-type selective alterations.

### 3.3. Compartmental Distribution of Chloride Transporters in Neurons

#### 3.3.1. Somatodendritic Compartment

Both NKCC1 and KCC2 are distributed differently across cellular compartments. NKCC1 mRNA is detected and abundant in most cells of the adult brain [21], but protein expression in mature neurons is low, which has hampered studying the subcellular distribution of this transporter.

Recently, a super-resolution microscopy study in dissociated neurons took advantage of hemagglutinin-tagged transporters in hippocampal neurons and showed that NKCC1 isoforms are detected in the somatodendritic compartment [54]. This study also found that the NKCC1-KCC2 ratio is higher in axon initial segments of hippocampal neurons (see Section 3.3.2). Another study found that in the adult brain, two KCC2 isoforms (KCC2a and KCC2b) are expressed, but their expression differs between different cellular compartments (E.G. soma and proximal dendrites versus distal neuronal dendrite [22]). Both dentate gyrus granule cells and CA1 pyramidal cells of the adult hippocampus show different patterns of KCC2 expression from distal/apical dendrites to the soma [55]. This observation also suggests that the NKCC1-KCC2 ratio is differentially regulated along dendrites, possibly providing local control over the efficacy of synaptic activation. Indeed, spatially nonuniform [Cl^−^]_i_ shifts have been described in dendrites [56]. In keeping with the idea of spatial segregation in [Cl^−^]_i_ gradients, KCC2 is enriched in the vicinity of synapses [57,58,59] by a gephyrin-dependent mechanism, suggesting that GABA_A_ is hyperpolarizing in these structures. In particular, KCC2 is clustered at the spine periphery rather than at the postsynaptic density (PSD) region [60]. NKCC1 is expressed in dendrites both at perisynaptic and extrasynaptic sites, where, in most cases, it colocalizes with KCC2 [54]. Intriguingly, these authors also showed that, in cultured neurons, NKCC1 and, to a lesser extent, KCC2 proteins are mobile, suggesting that their distribution can be rapidly modified in response to neuronal activity and the activation of individual synapses. This observation suggests that activity-dependent alterations in NKCC1/KCC2 activity contribute to synaptic plasticity. In agreement with this suggestion, a study showed that in hippocampal neurons (both cultured and in acute slices), KCC2 activity is regulated on a timescale of minutes by coincident postsynaptic spiking and synaptic activity by a calcium-dependent mechanism, which leads to persistent changes in synaptic strength [61].

#### 3.3.2. Axonal Structures

In mature neurons, NKCC1 appears to be more abundantly expressed in the axon initial segment (AIS) compared with the somatodendritic compartment, which creates an axo-somatodendritic gradient in intracellular chloride [62]. Additionally, KCC2 expression in the AIS is low [55,63] or absent [54]. This high NKCC1-to-KCC2 ratio maintains high [Cl^−^]_i_ and, therefore, a depolarizing effect of GABA_A_ in these structures. Accordingly, elegant work showed that the GABAergic input from axo-axonic cells to cortical pyramidal neuron axons is excitatory and elicits action potential firing in postsynaptic axons [63], although it may become hyperpolarizing in older animals [64].

### 3.4. Circadian Regulation of GABA_A_ Reversal Potential

A very recent paper reported another intriguing aspect of [Cl^−^]_i_ homeostasis in the adult brain, circadian regulation [65]. These authors showed that in the pyramidal neurons of the adult mouse neocortex baseline, [Cl^−^]_i_ undergoes a two-fold increase from day to night and that such an increase is due to changes in the surface expression and phosphorylation of NKCC1 and KCC2. These findings add another layer of complexity to the understanding of GABAergic signaling in the brain and provide further proof of the remarkable flexibility of this system.

## 4. Regulation of Neuronal GABA_A_ Reversal Potential in Disease

### 4.1. Depolarizing GABA_A_ Current

**Figure 1:**
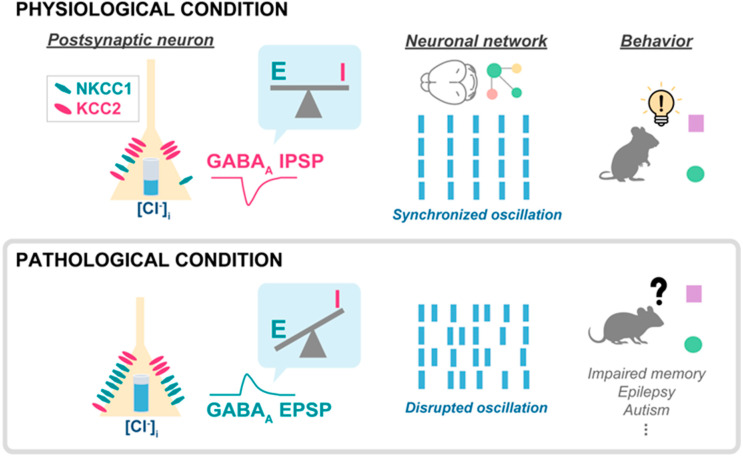
Schematic of the effects of depolarizing GABA_A_ in disease. In postsynaptic neurons, two types of chloride/potassium cotransporters (NKCC1 and KCC2) regulate intracellular chloride concentration ([Cl^−^]_i_). The polarity of the GABA_A_ current, either depolarizing or hyperpolarizing, is largely determined by the transmembrane chloride concentration gradient. When activation of GABA_A_ receptors pathologically evokes excitatory (EPSP) instead of inhibitory (IPSP) synaptic potentials due to increased NKCC1-KCC2 activity ratio, the excitatory/inhibitory (E/I) balance is altered, which leads to a disruption of network oscillations and behavioral impairment.

An altered NKCC1-KCC2 activity ratio has been linked with various neuropathological disorders and discussed in previous reviews [66,67]. Depolarizing GABA_A_ currents in the adult brain have been reported in many pathological conditions such as epilepsy [37,68], Down syndrome [69], schizophrenia [51], and severe stress [70]. The mechanism by which depolarizing GABA currents interferes with brain activity is likely caused by a loss of synchronization in network oscillations (Figure 1). The cellular mechanisms leading to the altered reversal potential include alterations in transporter expression and in their regulation. For example, increased activity of kinase, such as OXSR1 and WNK3, which regulate NKCC1 and KCC2 was reported in the prefrontal cortex of schizophrenia patients [71]. On the other hand, in subchronic phencyclidine (scPCP)-treated mice, an animal model that mimics the cognitive symptoms associated with schizophrenia [72,73], depolarizing GABA_A_ appears in the prefrontal cortex as a consequence of increased NKCC1 mRNA expression [51]. In line with these data, a gain-of-function NKCC1 mutation has been identified in human schizophrenia patients [74]. Thus, similar to the scenario in Down syndrome [69] and in the spinal cord of neuropathic pain models [75], NKCC1 is the transporter whose activity is mostly altered in schizophrenia. Therefore, the cellular mechanism leading to altered GABA_A_ reversal potential in the diseased adult brain appears to be different than in the neonatal brain, where the depolarizing GABA_A_ current is due to low KCC2 expression [39,40]. Importantly, the pharmacological inhibition of NKCC1 rescues cognitive impairment in both scPCP and Down syndrome mice. Additionally, depolarizing GABA has also been suggested as a mechanism leading to a loss of the synapse specificity of LTP in aging animals [76]. Finally, depolarizing GABA may also play a role in Alzheimer’s disease, as it was recently shown that NKCC1 expression is increased in the hippocampus of mice injected with amyloid β-peptide [77]. These authors further showed that treatment with beta-amyloid (Aβ1–42) also increases NKCC1 expression in hippocampal cultures. Another group [78] obtained complementary results showing KCC2 downregulation in 5xFAD mice, a widely used animal model of Alzheimer’s disease. Importantly, these authors also showed that treating the mice with the KCC2 activator CLP290 rescued cognitive performance.

### 4.2. Hypernegative GABA_A_ Current

**Figure 2:**
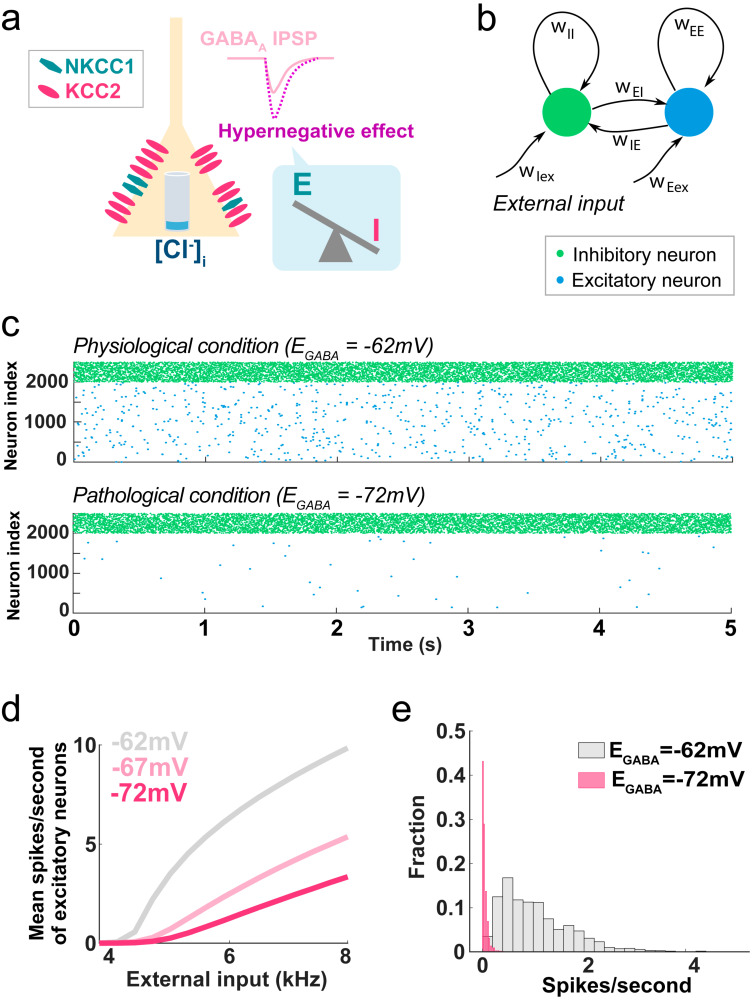
Effect of hypernegative GABA_A_ current on network function. (**a**) When the neuronal KCC2-NKCC1 activity ratio is increased, intracellular chloride concentration ([Cl^−^]_i_) is lower than the physiological level, and the inhibitory effect of GABA_A_ current is strengthened, disrupting the E/I balance. (**b**–**e**) Simulated effects of hypernegative GABA_A_ current on cortical network activity. In pathological conditions (GABA_A_ reversal potentials = −72 mV), the spike frequency of the postsynaptic excitatory neurons is severely decreased, even when the frequency of the inhibitory inputs is unaffected. (**b**) Computational model of a cortical network based on local microcircuit of excitatory–inhibitory recurrent architecture. (**c**) Simulated spontaneous spiking activity of excitatory (blue) and inhibitory (green) cortical neurons with physiological (−62 mV, top panel) or pathologically hyperpolarized (−72 mV, bottom) GABA_A_ reversal potentials. (**d**) Average firing rate of excitatory neurons with three different GABA_A_ reversal potentials in response to variable external input frequencies. (**e**) Histogram of firing rates of excitatory neurons when the external input was set at 4.5 kHz. (Adapted with permission from [16]).

So far, the study of GABA_A_ current reversal potential alterations in pathological conditions has been largely focused on the (re)appearance of excitatory GABA_A_ in adult animals. However, recent investigations have shown that the opposite regulation can also be detrimental and that excessive GABA_A_-mediated inhibition due to hypernegative current reversal potential represents another potential mechanism of pathology. For example, adult mice that were exposed to neonatal inflammation (using an LPS injection) show cognitive impairments associated with hypernegative GABA_A_ reversal potential and increased KCC2 activity in the hippocampus [79]. Interestingly, these authors showed a complex TGF-β1-dependent regulation, suggesting the alteration is not simply due to an overdrive of the normal developmental KCC2 upregulation. Accordingly, we recently showed that a hypernegative GABA_A_ current can appear in the adult brain independently of any developmental dysregulation. We found that in adult mice 7 days after peripheral nerve injury (the Spared Nerve Injury model of neuropathic pain), the reversal potential of the GABA_A_ current in the pyramidal neurons of the medial prefrontal cortex is ~10 mV more negative than in sham-operated control mice. This change is due to a large overexpression of KCC2 transcripts and, to a minor extent, to decreased NKCC1 expression [16]. Computational modeling of a cortical network demonstrated that the ~10 mV shift in GABA_A_ reversal potential is by itself sufficient to dramatically reduce the overall activity of the network, resulting in over 60%-decreased activity of excitatory neurons in the range of natural external inputs (Figure 2). Thus, hypernegative GABA has a major impact on neuronal activity and should be considered whenever increased inhibition is present and no changes in the activity and/or number of interneurons are detected.

### 4.3. GABA_A_ Flexibility and Pathology

Why are changes in GABA_A_ current reversal potential detected in so many different conditions? The proximity of the value of the GABA_A_ current reversal potential and the resting membrane potential of most neurons is probably the critical factor. Such proximity means that even small changes in the intracellular chloride concentration can switch the current effect from hyperpolarizing to depolarizing; this is a unique property of the GABA_A_ channel and sets it apart from other synaptic receptors. Another reason might be the intrinsic complexity and plasticity of the regulatory mechanisms. Intracellular chloride concentration changes significantly during development because the regulating machinery is heavily modulated by numerous mechanisms (including epigenetic [80]); this makes this process particularly sensitive to even small functional abnormalities. Therefore, we hypothesize that alterations in GABA_A_ reversal potential (either in the depolarizing or hyperpolarizing direction) in the cerebral cortex represent a common maladaptive mechanism leading to cognitive impairment in multiple pathological conditions with different etiologies.

## 5. Network Effects and Clinical Implications of Flexible GABA_A_ Reversal Potential

Numerous studies show that neuronal oscillations in the prefrontal cortex and hippocampus are closely associated with cognitive tasks. Theta rhythms (4–8 Hz) in the cortex are enhanced during memory tasks in humans and animals [81,82]. Gamma oscillations (25–100 Hz) are also critical for cognitive function, and aberrant gamma oscillations have been observed in multiple cognitive disorders. Additionally, changes in brain oscillations can predict cognitive symptoms in individuals with autism spectrum disorders, and increasing evidence shows that long-range gamma synchronization is reduced in patients with schizophrenia [83] and other conditions characterized by cognitive disability [84]. GABAergic inhibition is essential to the generation of oscillatory networks [85,86]. Accordingly, E/I balance in the PFC and hippocampus is critical for cognitive and social functions [87,88]. However, despite the centrality of E/I imbalance in numerous brain disorders, the cellular mechanisms of E/I imbalance are mostly unknown. As oscillations emerge from reciprocal interactions between populations of excitatory (glutamatergic) and inhibitory (GABAergic) neurons [89], E/I imbalance may be due to multiple types of abnormalities in GABAergic and/or glutamatergic signaling. Consequently, it is possible that differences in the mechanisms underlying E/I imbalance lay behind the large variety and diverse severity of cognitive impairments in different neurodevelopmental disorders. While abundant evidence has been collected over the past several years supporting the role of depolarizing GABA as an important pathogenic mechanism in various diseases, it has only become clear very recently that the hypernegative reversal potential of the GABA_A_ current also has a major functional impact, although the network mechanisms are likely different. We suggest that the main functional impact of depolarizing GABA is a loss of network synchronization, which results in the disruption of network oscillations (Figure 1). On the other hand, a hypernegative GABA_A_ current seems to cause a general inhibition of the circuit, roughly equivalent to an increased number of inhibitory synaptic inputs (Figure 2).

Additionally, while both these conditions represent dysfunctions in GABAergic signaling, their potential treatment requires completely different approaches, each with potential drawbacks. The case of hypernegative GABA_A_ is theoretically straightforward because GABA antagonists are likely beneficial to reduce the effects of the hypernegative GABA_A_. The use of such drugs, however, is limited by their toxicity and epileptogenicity. The situation is more complex in the case of depolarizing GABA, where inhibition is insufficient but cannot be improved by the common approach of using GABA agonists such as benzodiazepines or GABA analogs such as valproic acid or gabapentin. The most direct treatment for this condition would obviously be the restoration of the inhibitory effect of GABA_A_, which can be achieved either pharmacologically or genetically [51,69]. Both these approaches, however, also have major limitations. As depolarizing GABA in disease conditions is, by and large, the consequence of NKCC1 upregulation, NKCC1 antagonists, such as bumetanide, may provide a treatment opportunity. However, these drugs have very poor BBB permeability [90], which limits their effective concentration in the brain parenchyma. Additionally, these drugs do not have any cellular selectivity and not only affect excitatory and inhibitory neurons but also non-neuronal cells, which often express NKCC1 at a higher level than neurons. Thus, the network effects are necessarily broad and hard to predict. The genetic approach may help as NKCC1 expression could be selectively repressed in select cellular populations, but this approach is still far from viable for clinical treatment. An alternative approach may take advantage of KCC2 agonists, which would be neuron-selective, but effective agonists are still missing. However, KCC2 activity is regulated by numerous signaling pathways such as WNK, PKC, and BDNF [20,71,91,92,93]; therefore, these might also represent interesting pharmacological targets. Even in this case, however, cell-type selectivity would help considerably.

## 6. Conclusions

The GABA_A_ current represents a particularly vulnerable target in many neurological conditions. This is mainly driven by two facts: 1—The net effect of GABA_A_R activation may be inhibitory or excitatory, depending on the electrochemical gradient for the permeant anions across the membrane of the postsynaptic neurons. Additionally, even when inhibitory, the GABA_A_ current may dramatically change its efficacy depending on its reversal potential. Consequently, this highly plastic and regulated system is sensitive to even small changes in transmembrane ionic gradients. 2—This functional balance is regulated by mechanisms that are highly plastic because they are designed to change during physiological development. Taken together, these factors explain both the particular vulnerability of this system to even small cellular alterations and the oversized functional effects that can derive from these alterations. In keeping with such strong vulnerability, alterations in GABA_A_ reversal potential have been recently implicated in numerous psychiatric and neurological conditions, suggesting that regulation of chloride-cation cotransporters represents a viable therapeutic target.
